# Is ventromedial prefrontal cortex critical for behavior change without external reinforcement?

**DOI:** 10.1016/j.neuropsychologia.2018.12.008

**Published:** 2019-02-18

**Authors:** Nadav Aridan, Gabriel Pelletier, Lesley K. Fellows, Tom Schonberg

**Affiliations:** a Department of Neurobiology, Faculty of Life Sciences and Sagol School of Neuroscience, Tel Aviv University, Tel Aviv 6997801, Israel; b Montreal Neurological Institute, Canada; c Department of Neurology and Neurosurgery, McGill University, Montreal, Canada

## Abstract

Cue-approach training (CAT) is a novel paradigm that has been shown to induce preference changes towards items without external reinforcements. In the task, the mere association of a neutral cue and a speeded button response has been shown to induce a behavioral choice preference change lasting for months. This paradigm includes several phases: after the training of individual items, behavior change is manifested in binary choices of items with similar initial values. Neuroimaging data have implicated the ventromedial prefrontal cortex (vmPFC) in the choice phase of this task. However, the neural mechanisms underlying the preference changes induced by training remain unclear. Here, we asked whether the ventromedial frontal lobe (VMF) is critical for the non-reinforced preference change induced by CAT. For this purpose, 11 participants with focal lesions involving the VMF and 30 healthy age-matched controls performed the CAT. The VMF group was similar to the healthy age-matched control group in the ranking and training phases. As a group, the healthy age-matched controls exhibited a training-induced behavior change, while the VMF group did not. However, on an individual level analysis we found that some of the VMF participants showed a significant preference shift. Thus, we find mixed evidence for the role of VMF in this paradigm. This is another step towards defining the mechanisms underlying the novel form of behavioral change that occurs with CAT.

## Highlights

• We tested participants with focal lesions involving ventromedial frontal lobe (VMF).
• Participants performed a behavioral change task without external reinforcements.
• The VMF group did not exhibit behavior change but some individuals in it did.
• We find mixed evidence for the role of VMF in this paradigm.

## 1 Introduction

Decision neuroscience has contributed to the understanding of maladaptive motivated behavior in conditions such as substance abuse, pathological gambling, and obesity (Bechara, 2005, Davis et al., 2004, Diekhof et al., 2008, Ernst and Paulus, 2005, Goudriaan et al., 2005). This knowledge offers opportunities for the development of new interventions to support behavior change. Most of the work in this area in humans has focused on the use of external reinforcement (Cahill and Perera, 2008, O’Doherty et al., 2004, Petry, 2010) and self-control (Baler and Volkow, 2006, Carden and Wood, 2018) to over-ride unwanted behaviors. Recently, Schonberg et al. (2014) introduced the Cue-approach training (CAT) task, in which merely associating an image with a cue and a speeded button press leads to long-lasting preference changes. The effect has been replicated in dozens of independent samples, showing changes in preferences for snack food items, unfamiliar faces, fractal art images and positive emotional images (Bakkour et al., 2017, Bakkour et al., 2016, Salomon et al., 2018, Zoltak et al., 2017). The CAT shows that an association of a neutral cue and a motor response with individual items (termed “Go” items) can change preference for different stimuli without external reinforcement or self-control, offering a novel avenue for addressing maladaptive choices.

The CAT procedure includes several phases. First, participants rank the stimuli to indicate their subjective preference. Based on the initial ratings, items are chosen to be associated with the button press and the cue in the subsequent training phase. During training, the entire stimulus set is presented on the screen several times with some of the items consistently associated with the cue and the button press (“Go” items). Then, preference change is probed in a binary choice phase where pairs of items of similar initial rankings are pitted against each other. If training did not influence preference, participants are expected to be indifferent between the two items (i.e. at chance). While prior behavioral studies in healthy young people have shown that CAT produces a replicable group effect of about 60–65% preference of the trained Go items, the cognitive and neural mechanisms underlying this effect remain unclear. Eye-gaze data during the probe phase showed greater gaze towards Go items even when they were not chosen, compared to No-Go items. This suggests that the induced shift of preference in the CAT relies on attentional mechanisms to transform the low level visual, auditory and motor features of the training into an updated value of the associated items. Functional magnetic resonance imaging (fMRI) studies of CAT have implicated the ventromedial prefrontal cortex (vmPFC) in the probe phase of the task, with greater activations in vmPFC for choices of Go items compared to choices of No-Go items modulated by the preference for individual items (Bakkour et al., 2017, Schonberg et al., 2014). In the original study (Schonberg et al., 2014), activation of vmPFC was also observed at the end of the training phase. However, similar activation was found for both Go and No-Go items. While these studies implicate the vmPFC in the CAT during the training and choice phases, they do not reveal whether this region plays a crucial role in this preference manipulation.

VmPFC and adjacent medial orbitofrontal cortex (mOFC; together termed ventromedial frontal cortex; VMF) have been implicated in representation and dynamic updating of value both in animals and humans (Wallis, 2012). In humans, activity within this area has been shown to scale with increasing subjective value across a range of stimulus types and tasks, and in some paradigms, to predict value-based choice (Bartra et al., 2013, Levy and Glimcher, 2012). Further evidence for the critical role of VMF in value-based decisions comes from lesion studies. Participants with VMF damage show impaired performance in flexible value learning tasks (Fellows and Farah, 2003, Tsuchida et al., 2010) and make less consistent preference judgments compared to healthy controls (Camille et al., 2011; Fellows and Farah, 2007; Henri-Bhargava et al., 2012). VMF damage was found to disrupt biasing of attention to rewarding features of the environment, suggesting that this area is critical to the interplay of attention and value in decision-making (Vaidya and Fellows, 2015). A recent study of reward incentivization in a speeded saccade task found that VMF damage reduced reward sensitivity, providing further causal evidence for the role of VMF in the evaluation of rewards (Manohar and Husain, 2016).

**Fig. 1:**
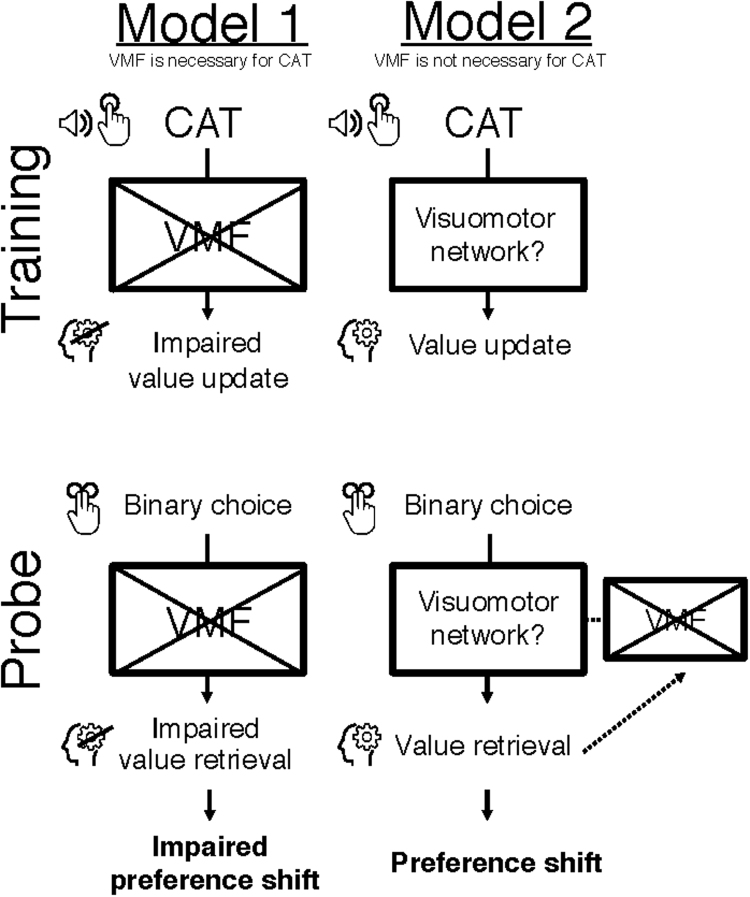
Putative models for the mechanisms underlying CAT.

Activation within vmPFC during choice in the fMRI studies of CAT suggests that this region might be necessary for the value update that underpins preference change in CAT. Alternatively, other structures (e.g. the visuomotor network) encode the value update and vmPFC is merely active during the choice of the preferred item, reflecting the updated value rather than making a causal contribution to the preference change. These two models propose different roles for vmPFC in CAT (Fig. 1); in the first, this region dynamically assigns credit following low-level attentional training. In the second, vmPFC is not involved in modifying value, but is involved in value representation during choice. These models make different predictions regarding the effects of VMF damage on CAT performance: if intact vmPFC is necessary for the CAT effect, individuals with VMF damage (i.e. affecting vmPFC) will show an attenuated or absent shift of preference following CAT. Alternatively, if vmPFC is not necessary for the value updating during training or value retrieval during choice, VMF damage will not affect preference shifts following CAT. In the current study, we tested these competing hypotheses by examining whether focal VMF damage affects the shift of preferences observed following CAT. Understanding the effects of VMF damage on behavior change with CAT will shed light on this novel non-externally reinforced procedure as well as more generally, on the role VMF plays in value construction and assignment during value based-decision making.

## 2 Materials and methods

### 2.1 Participants

Participants with focal lesions involving the orbitofrontal cortex (OFC) and ventromedial prefrontal cortex (vmPFC), together referred to here as ventromedial frontal: VMF (*N* = 11, mean age = 59.4 [39–78] years, 5 males), were recruited from the Cognitive Neuroscience Research Registry at McGill University. All had fixed, circumscribed lesions of at least 6-months duration (mean duration = 9.3 [5.4–16.5] years). Lesions were due to ischemic stroke, tumor resection, or aneurysm rupture. Thirty age-matched healthy control participants were recruited through local advertisements in Montréal. They were free of neurological or psychiatric disease and were not taking any psychoactive drugs. One control participant was excluded from the analysis due to extremely inconsistent choices (choice prediction accuracy = 0.52 z = –3.62; see Results for details). For the 29 included in this group, the mean age was 60.5 [44–79] y and 15 were females. All participants provided written, informed consent in accordance with the Declaration of Helsinki and were paid a nominal fee for their time. The study protocol was approved by the local Research Ethics Board. Table 1

**Table 1:** VMF group neuropsychological screening test performance [mean (SD)].

	Incidental memory (accuracy)	Fluency – animals (words/1 min)	Fluency – F (words/1 min)	Backwards digit span	Sentence comprehension (accuracy)
VMF _N = 11_	0.81 (0.14)^a^	17.6 (2.4)	10.6 (5.6)	2.8 (0.9)	0.99 (0.02)^a^

a Missing data from 1 subject.

### 2.2 Lesion analysis

**Fig. 2:**
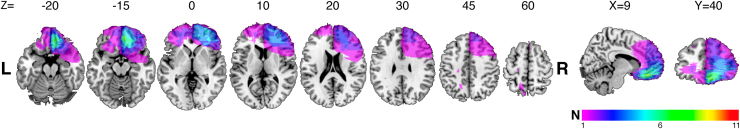
Lesion extent and overlap. Lesion overlap across the VMF participants is indicated by color bar, MNI brain slice coordinates are indicated by XYZ.

Individual lesions were traced from the most recent clinical computed tomography or magnetic resonance imaging onto the standard Montreal Neurological Institute (MNI) brain using MRIcro software (Rorden and Brett, 2000; www.mccauslandcenter.sc.edu/mricro/) by a neurologist experienced in imaging analysis and blind to task performance. MRIcron (www.nitrc.org/projects/mricron) was used to generate lesion overlap images (Fig. 2).

### 2.3 Procedure

Sixty identically-sized color images of computer-generated fractal art images served as the stimuli (“Fantastic Fractals, ”, 2013). The experiment was run using MATLAB (Mathworks, Inc. Natick, MA, USA) on a 21-in. screen.

#### 2.3.1 Binary ranking

**Fig. 3:**
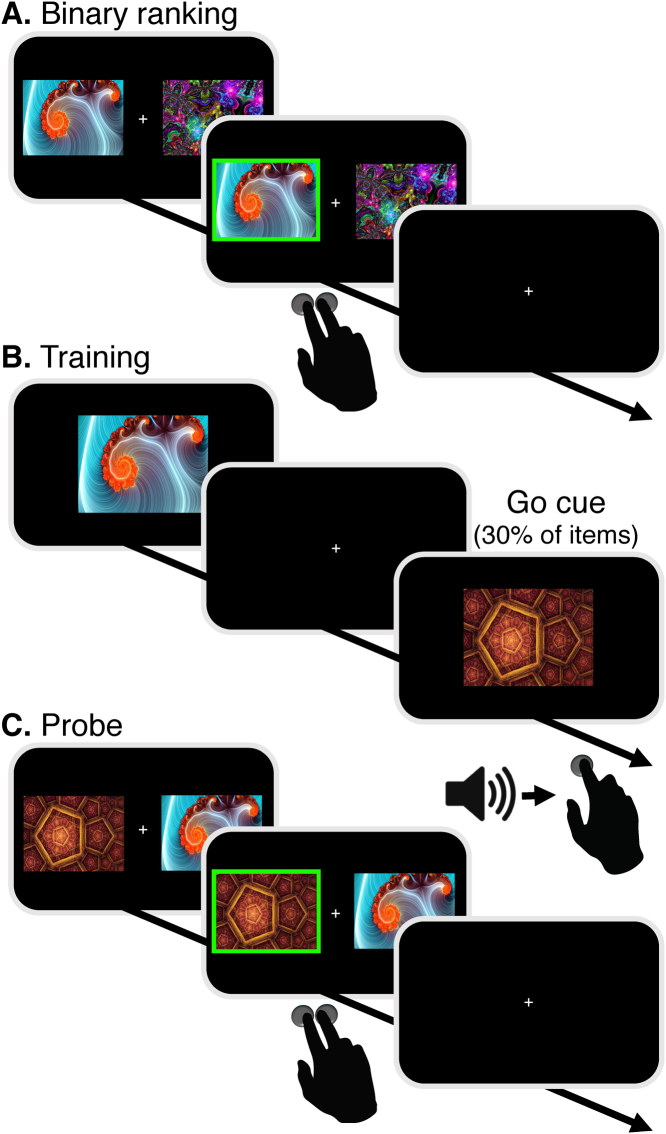
Procedure. A) Binary choices between pairs of 60 fractal images were used to obtain rankings; B) Training of Go-items, consistently paired speeded button presses cued by a neutral tone across 16 runs; C) Probe: binary choices between pairs of Go and No-Go items with similar initial ranking.

A forced-choice binary ranking procedure was used to estimate participants’ baseline subjective preferences for each of the stimuli. In this task, 60 stimuli were randomly paired to form 300 unique pairs. For each pair of stimuli, participants had 2500 ms to choose their preferred stimulus, followed by a 500 ms choice confirmation screen and 500 ms fixation cross (Fig. 3A). Based on the assumption of choice transitivity from rational choice theory (Regenwetter et al., 2011, Morgenstern and Von Neumann, 1953), we used the outcomes from the set of binary choices to infer individual preferences for the presented set of stimuli. That is, if stimulus A is preferred over B and stimulus B is preferred over C, then their respective ranks are A≻B≻C. We used the Colley Matrix algorithm (Colley, 2002), designed to solve ranking problems with limited number of binary outcomes to maximize ranking validity and specificity. This procedure resulted in a ranked list of the 60 stimuli, based on each participant's individual preferences. Colley Matrix ranking scores typically range from 0 (least liked) to 1 (most liked), with a fixed mean of 0.5. An intransitive choice pattern is characterized by densely distributed scores around the center of 0.5, while a distinct preference pattern leads to more distributed ranking scores. From these rankings, we quantified a transitivity score for each participant as the standard deviation of the participant's ranking scores.

#### 2.3.2 Cue-approach training

Following the baseline evaluation of subjective preferences using the binary ranking procedure, participants underwent 16 training runs of the cue-approach training procedure. A subset of 40 stimuli, consisting of 20 high-value (ranked 3–22, above the median rank) and 20 low-value items (ranked 39–58) were displayed once during each run. Twelve items from the subset (6 high-value, mean rank = 12.5 and 6 low-value mean rank = 48.5) were consistently associated with a Go auditory-cue (Fig. 3B). Participants were instructed to respond to the Go cue by pressing a keyboard button as fast as possible, before stimulus offset. Participants were not informed in advance that the association of stimuli with the cue will be consistent or which items would be Go items and no feedback was given. Each stimulus in the training set was presented individually on the screen for 1000 ms after which it was replaced by a fixation cross. The cue appeared following an adaptive staircase according to each participant's performance, as in previous CAT studies (Schonberg et al., 2014), this ensured maintaining a similar difficulty level throughout the training phase and across individuals. Stimuli were randomly ordered and followed by a jittered fixation cross with an average duration of 2000-ms (range of 1000–6000 ms, 1000 ms intervals). A short demonstration was included before the beginning of the full training phase in order to introduce the auditory Go cue and ensure the participants understood the instructions correctly.

#### 2.3.3 Probe

Preference change following CAT was evaluated in a probe phase. On each probe trial, two items appeared to the right and left of a central fixation cross and participants were asked to select their preferred stimulus. In each pair, both items were of similar initial value (either high-value or low-value), but only one item was a Go item, i.e. associated with a cue during training. For each pair, participants had 1500-ms to select their preferred stimulus, followed by a 500-ms choice confirmation and a fixation cross for a jittered duration with an average of 3000-ms (range of 1000–11000 ms, 1000 ms intervals; Fig. 3C). In addition to these comparisons, as in previous CAT experiments, ‘sanity check’ trials were also incorporated in the probe phase to measure preferences consistency. In the ‘sanity check’ trials, participants were asked to choose between pairs of items in which one item was of initial high-value and the other of initial low-value (both Go or both No-Go items), to validate the stability across time of the initial preference evaluation. The probe phase included two runs with 152 total trials, with all unique probe pairs presented in a random order in each run.

#### 2.3.4 Memory

At the end of the experiment, participants performed two sequential memory tasks. The first assessed memory for fractals presented during the experiment compared to novel items (Old/New). The second assessed whether participants remembered which images were associated with the cue (Go/No-Go).

### 2.4 Statistical analyses

Binary choice outcomes were analyzed using a mixed-model logistic-regression (R package lme4 v1.1–13). Group means were compared by a two-sample permutation test (R package Deducer v0.7–9), and 95% confidence intervals were estimated using data bootstrapping.

Voxel level lesion analysis was used to test for associations between lesion location and CAT effect. For every voxel in which at least three VMF participants had damage, a permutation test was used to calculate the statistical significance of the difference in Go item choice ratio in the probe phase between VMF participants that had damage within that voxel and those who did not. Images of the results of this analysis were created using the software MRIcroGL (https://www.mccauslandcenter.sc.edu/mricrogl/home).

### 2.5 Data sharing

Behavioral data and analysis codes are available at osf.io/d8ceg/.

## 3 Results

See Table 2 for summary of behavioral results for all tasks.

**Table 2:** Behavioral results [mean (SD)].

			**control**	**VMF**	**p**
Binary ranking	choice consistency (%)	80.5 (5.9)	80.5 (6.3)	0.95	
	choice RT (ms)	1197 (215)	1329 (129)	0.026^a^	
	Inconsistent-consistent choice ΔRT (ms)	168 (117)	194 (97)	0.47	
	Mean correlation of choice-difficulty and RT	−0.29 (0.13)	−0.32 (0.10)	0.56	
Training	cue RT (ms)	377 (73)	393 (89)	0.29	
	commission error rate (%)	0. 4 (0. 4)	0. 4 (0. 4)	0.82	
	omission error rate (%)	0. 5 (1. 2)	0. 2 (0. 3)	0.19	
Probe	Go/No-Go choice (%)	59.3 (13.6)	50.3 (15.8)	0.11	
	choice RT (ms)	836 (105)	931 (91)	0.016^a^	
	preferences consistency (%)	91.2 (13.6)	95.4 (6.3)	0.28	
Memory	experiment items recognition (was/was not; %)	91.7 (8.7)	95.4 (3.7)	0. 26	
	RT (ms)	1208 (525)	1041 (188)	0.29	
	training condition recognition (Go/No-Go; %)	64.1 (17.5)	56.4 (14.2)	0.20	
	RT (ms)	2009 (1292)	1602 (776)	0.15	

a < 0.05.

### 3.1 Binary ranking

**Fig. 4:**
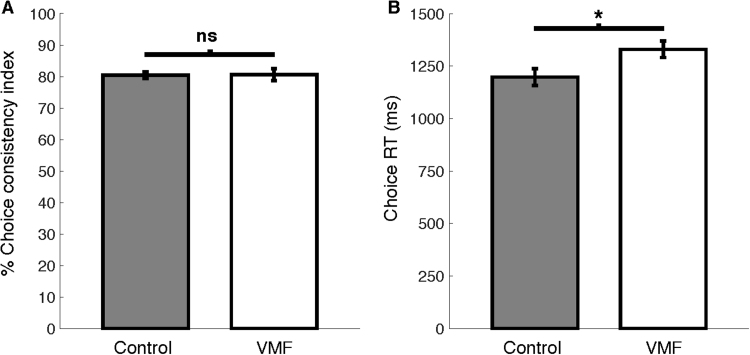
Binary ranking. A) There was no difference in choice consistency between the control and VMF groups B) The VMF group took longer time to make choices. Error bars represent standard error of the mean.

For each participant, we estimated choice consistency as the prediction accuracy of a “leave one out” model. For each of the 300 binary choices, we applied the Colley-matrix algorithm to the remaining 299 choices, which yielded the ranks of the 2 competing items in this trial. For each participant, we defined the choice consistency index as the proportion of trials in which the highest ranked item was chosen (% correct choice predictions). We did not find a difference in choice consistency between the groups (t_(16.9)_ = −0.08, p = 0.96, 95% CI [−4.5, 4.1]; Fig. 4A). We found a difference between the control and VMF groups in the mean reaction time during binary choices such that overall, the VMF group took slightly longer to make choices (t = −2.37, p = 0.026, 95% CI [−239, −25]; Fig. 4B). Examining the difference in RT of consistent and inconsistent choices, we did not find a difference between the control and VMF groups (t_(30.1)_ = 0.70, p = 0.47, 95% CI [−45, 96]).

We next examined the relationship between choice-difficulty (items’ rank difference) and reaction time. In both groups, there was a negative correlation between the rank difference of a given pair of items and choice reaction time, such that the larger the rank difference between the two items, the faster the choice was made. This correlation was similar in control and VMF groups (r_control_ = −0.29, r_VMF_ = −0.32, t_(21.6)_ = 1.26, p = 0.29, 95% CI [-0.05, 0.09]).

### 3.2 Cue approach training

There were no differences between the groups in the button-press reaction time to the tone cue (calculated as time after cue) during training (t_(15.3)_ = 1.26, p = 0.29, 95% CI [−73, 41]). Similarly, there were no differences between the groups in commission error rate (t_(19.3)_ = −0.17, p = 0.82, 95% CI [−0.003, 0.002]) or omission error rate (t_(34.3)_ = 1.35, p = 0.19, 95% CI [−0.001, 0.007]) during training.

### 3.3 Probe

#### 3.3.1 Group analysis

To assess preference changes following training, we analyzed the proportion of probe trials in which participants preferred the Go items over the No-Go items, using a two tailed repeated measures logistic regression. In each pair, both items were of similar initial preference based on the baseline evaluation phase. As in previous studies, we hypothesized that the cue approach effect would enhance preferences for the Go items above the chance level of 50% of trials (log-odds = 0; odds-ratio = 1).

**Fig. 5:**
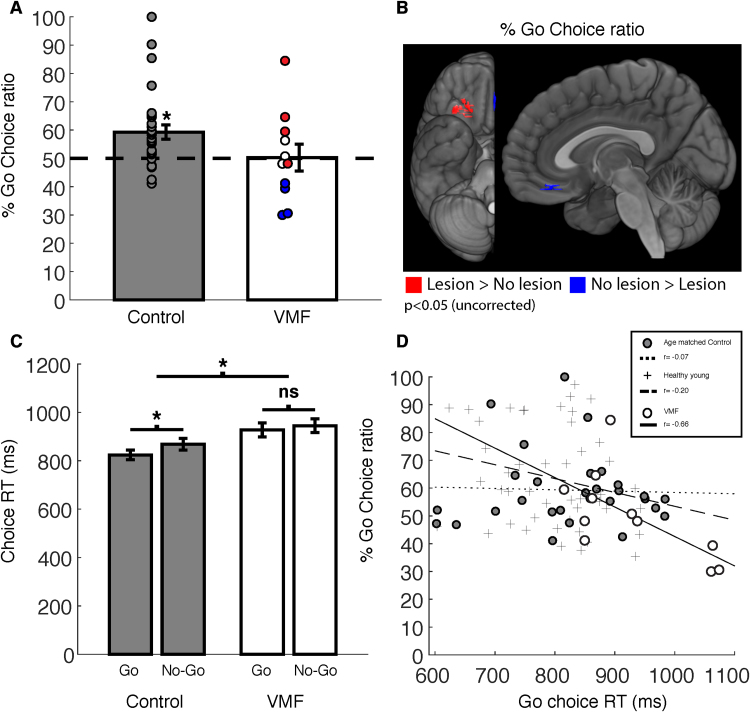
Probe. A) Ratio of Go to No-Go choices. B) Voxel level lesion analysis. Participants with vmPFC damage chose Go items less than participants with intact vmPFC (blue) and participants with posterior-central OFC damage chose Go items more than participants with intact posterior-central OFC (red). C) Probe choice reaction time. D) Correlation across participants between Go choice-time and ratio of Go to No-Go choices.

Following cue-approach training, the control group showed increased preference for the Go items over the No-Go items during probe (mean proportion = 59.28% (13.6), odds ratio (OR) = 1.58, 95% CI [1.17, 2.16], p = 0.002; Fig. 5A). In the VMF group, participants did not show a preference for Go over No-Go items during probe (mean proportion = 50.39% (15.8), OR = 1.03, 95% CI [0.664, 1.59], p = 0.896; Fig. 5A, see Fig. 5B for individual VMF participants lesion maps and color codes). The difference between the control and VMF groups in Go vs. No-Go choice proportion was not significant (Δ_(control-VMF)_ mean proportion = 8.89%, OR = 0.652, 95% CI [0.378, 1.12], p = 0.11).

#### 3.3.2 Individual participant analysis

For each participant, we calculated the individual probability of obtaining a preference shift. We defined the threshold of individual learning based on the binomial distribution compared to chance: P_random choice probability_= 0. 5, $\frac{K\mathrm{Go choices}}{N\mathrm{probe binary choices}}\left(\mathrm{for critical z of p}<0.05\right)$ = Go choice-ratio ≥ 57.64%. This resulted in three of the 11 VMF participants and 12 of 29 participants in the control group showing Go choice-ratio larger than the critical Go choice-ratio. Four VMF participants were below chance, compared to one in the control group (Go choice-ratio ≤ 42.36%).

#### 3.3.3 Voxel level lesion analysis

We found two distinct clusters in which Go vs. No-Go choice proportion was different between VMF participants. Participants with damage that included a region within vmPFC chose the Go items less than VMF participants with that sub-region spared. In contrast, those in whom damage affected the right posterior-central OFC chose the Go items more than VMF participants with that region intact. Caution is needed in interpreting these results, as the sample size is small for a voxel-based analysis, the power to detect effects is limited and not homogeneous across VMF and results are not corrected for multiple comparisons.

#### 3.3.4 Reaction time during probe

We found a difference between the control group and VMF group in choice reaction time during the probe phase with longer RTs for the VMF group (t_(20.7)_ = −2.79, p = 0.011, 95% CI [−160, −32]). In the control group the choice time of the Go items was faster than choice time of the No-Go items (t_(28)_ = -2.88, p = 0.008, 95%, CI [-75, −13]). In the VMF group there was no difference in choice time of the Go and No-Go items (t_(10)_ = −1.28, p = 0.23, CI [−47, −13]; Fig. 5C). However, the interaction of group (control/VMF) and item (Go/No-Go) was not significant (p = 0.29). Choice reaction time and preference are known to be linked (Mir et al., 2011, Shadlen and Shohamy, 2016). Considering the difference between the control and VMF groups in choice reaction time in the probe, we explored the possibility that Go items’ choice time may reflect individual differences in the degree of preference shift following CAT. Across participants, we fitted a linear regression between choice reaction-time of Go items and the degree of preference shift toward the cued Go items. In the control group there was no correlation (N = 29, r = −0.04, p = 0.84). However, in the VMF group, we found a significant negative correlation (N = 11, r = -0.64, p = 0.03) such that the faster the participant chose the Go items the greater the ratio of Go over No-Go items chosen. For further validation of this comparison, we examined this correlation in previously collected data of a similar procedure in healthy young adults (Salomon et al., 2018, experiments 2 and 8). Similar to the control group of our study, there was no correlation between Go choice RT and preference shift (N = 49, r = −0.20, p = 0.17; Fig. 5D).

#### 3.3.5 Probe preference consistency

There were no differences between the groups in preference consistency (ratio of choosing high value items to low value items) during probe between the groups (t_(36.2)_ = −1.35, p = 0.28, 95% CI [−0.10, 0.02]).

### 3.4 Memory

At the end of the experiment, we tested participants’ memory of the items (were the items presented in the experiment, or novel) and of the training condition for the items (were the items associated with a cue and button-press response, or not). We did not find a difference in the proportion of items recognized between the control and VMF groups (t_(37.3)_ = −1.48, 95%, p = 0.28, CI [−8.7, 3.7]). We did not find a difference in the recognition ratio of the experiment items’ training condition between groups (t_(15.3)_ = 3.92, p = 0.29, 95% CI [−3.3, 18.5]). There was no difference between the control and VMF in the reaction time to recognition of the experiment items (t_(38)_ = 1.47, p = 0.29, 95% CI [−52, 384]), and no difference between the control and VMF in the reaction time to recognition of the experiment items’ training condition (t_(22.8)_ = 1.15, p = 0.38, 95% CI [−251, 1064]).

## 4 Discussion

In this study, we examined whether VMF is critical for behavior change that does not rely on external reinforcement in the cue approach task. We aimed to differentiate between two potential underlying models of the effect: in one, VMF has a crucial role in the transformation of the visual, auditory and motor features of training into an enhanced value of the cued items. In the other, preference change with CAT does not critically depend on VMF. We tested these competing models by studying participants with VMF damage and healthy age-matched control and examined performance across the different phases of the CAT task. We found that the control group showed a significant preference shift following CAT, while the VMF group did not. However, the difference between the control and VMF groups in the magnitude of this shift was not significant.

The CAT effect is reliably evident at the group level, but vary at the individual level (Bakkour et al., 2017, Bakkour et al., 2016, Schonberg et al., 2014, Veling et al., 2017, Zoltak et al., 2017). There was greater variability in the CAT effect within the VMF group, notably including 4/11 individuals who showed a systematic preference shift towards the ‘No-Go’ items, a pattern seen in only 1/29 controls. It is possible that these opposing patterns of the individuals within the VMF group yielded an absence of the group effect. In addition, since the range for observing a reduced CAT effect is relatively narrow (50–58% Go choices), detection of such an effect could be challenging (i.e. a floor/ceiling effects).

By design, all the participants in our study had lesions that affected VMF, but the damage extended to varying degree into ventrolateral prefrontal cortex or the frontal pole. In addition, the VMF encompasses several anatomically and functionally distinct sub-regions (Rudebeck et al., 2008). We have previously observed different deficits in reward learning processes in patients with predominantly medial compared to lateral VMF damage (Noonan et al., 2017). Manohar and Husain (2016) found that a group with VMF damage showed an overall decreased sensitivity to reward, but some participants within that group showed an opposite effect. It is possible that in our study variation in the sub-regions damaged within the VMF explain the variation in CAT effect. The sample size is too small to provide a rigorous test of this speculation, but a preliminary voxel-based analysis suggested that damage to vmPFC was associated with the atypical ‘opposite CAT effect’, i.e. a preference shift away from the Go-trained items (Fig. 5B). Previous fMRI studies of CAT found that the vmPFC had greater activations for choices of Go items compared to choices of No-Go items during the binary choice phase (Bakkour et al., 2017, Schonberg et al., 2014). The evidence of an opposite CAT effect in participants that had damage within this region fits with these imaging findings that implicated vmPFC in preference change following CAT. It should be noted, we did not predict a systematic shift in the opposite direction with vmPFC damage. In the second resulting area, damage within the posterior-central OFC was associated with higher choice of the Go items. This finding does not allow to determine whether there is a link between OFC and CAT effect, but may serve as a basis for future hypotheses.

As a group, the VMF participants performed similarly to the healthy aged-matched control in most other phases of the task. VMF participants were indistinguishable from controls in the training phase, showing intact ability to rapidly respond with a button press to the auditory cue. During the binary ranking, the VMF participants were able to make consistent preference-based choices between abstract fractal images, similar to the control group. This finding supports previous reports of consistent value-based ratings and choices of artwork stimuli in individuals with VMF damage over short time frames (Vaidya and Fellows, 2015). In contrast, several studies reported that individuals with VMF damage were less consistent in choices of various stimuli including food items, images of human, animals and landscapes and colors (Camille et al., 2011; Fellows and Farah, 2007; Henri-Bhargava et al., 2012) and impaired in transitive (Koscik and Tranel, 2012) and associative inference-learning (Spalding et al., 2018). In our study, we found no differences between the VMF and control groups in choice consistency during the binary ranking phase and over the course of the 1 h experimental session. Together with the wider literature, this suggests that VMF is required for consistent valuation of only certain categories of stimuli, or under specific experimental conditions. The mechanisms underlying this observation remain to be clarified but may relate to a role for VMF in flexibly prioritizing attributes during evaluation of complex stimuli (Vaidya et al., 2017).

Interestingly, we found that the VMF participants were somewhat slower to make choices (regardless of choice difficulty or choice consistency with overall individual preference) both in the binary ranking phase and in the probe phase compared to the control group. While both groups showed a systematic relationship between RT and choice difficulty (i.e. value difference), as we have seen in other preference paradigms (Henri-Bhargava et al.), only the control group was faster to select Go items than No-Go items. There are conflicting reports regarding choice reaction-times and VMF damage. Rogers et al. (1999) found longer deliberation time in participants with damage to OFC compared to participants with damage to dorsal or lateral prefrontal-cortex and healthy controls in a decision task that involved economic choices with varying risks and monetary rewards. Other work using naturalistic stimuli (people, objects) reported choice reaction time was unaffected by VMF damage (Fellows and Farah, 2007).

A recent study showed that allowing participants extra time to make a choice eliminated the CAT effect (Veling et al., 2017). When we explored whether choice time of Go items could explain the variability of the Go to No-Go choice ratio here, we found a correlation only in the VMF group. Given the small sample size, the strength of this correlation between cued items choice time and the preference shift should be treated with caution. Nevertheless, the existence of a significant correlation supports the possibility that longer choice times in the VMF group (not seen in controls) contributed to the lack of a group effect. Further investigation is required to fully determine how VMF damage, decision time and preferences are linked.

Beyond our main aim to shed light on the neural mechanism of CAT, this study is novel in several additional aspects. First, we show for the first time that a sample of healthy elderly participants (mean age 60.5) express a CAT effect (at the group level), similar to that observed in previous samples of young healthy adults (mean age 24; Salomon et al., 2018). Second, we provide further support to previous findings that people with VMF damage can make consistent preference judgements, at least under specific conditions. We adopted the triangulation approach to study the neural basis of a novel non-reinforced behavioral change task by combing evidence from previous behavioral and imaging studies with the advantages of a lesion study (Munafò and Davey Smith, 2018). However, the findings do not allow to determine whether intact VMF is critical to induce non-externally reinforced preference change, by the mere association of cues and button presses. The evidence linking different CAT effects with damage to different regions within the VMF and the correlation between reaction times and the degree of effect in the task suggest fruitful directions for future work. Further research is needed to fully determine the underlying neural mechanisms of the CAT and the role value representations within the VMF are playing within it. Finally, understanding the basis for individual differences in CAT effects will be important in motivating and designing trials of CAT as a novel intervention for behavior change.
